# May I have your attention, please? An investigation on opening effectiveness in e-mail marketing

**DOI:** 10.1007/s11846-022-00517-9

**Published:** 2022-04-20

**Authors:** Julián Chaparro-Peláez, Ángel Hernández-García, Ángel-José Lorente-Páramo

**Affiliations:** Departmento de Ingeniería de Organización, Administración de Empresas y Estadística. ETSI de Telecomunicación, Universidad Politécnica de Madrid, Av. Complutense 30, 28040 Madrid, Spain

**Keywords:** E-mail marketing, Digital marketing, Effectiveness, Open rate, Attention, Online communication, M37 Advertising, M31 Marketing

## Abstract

Academic research has yet to provide a comprehensive view on how to capture individuals’ attention when a promotional e-mail reaches their inbox. This study investigates the variables that influence consumers’ attention toward promotional e-mails, operationalized as open rates, and proposes an integrative model that combines and integrates visible, temporal, and contextual elements. The empirical analysis uses ordinary least squares linear regression to validate the model with data obtained from a multinational sample. The dataset, which is global in nature, comprises 5765 different promotional e-mails sent between 2013 and 2018 by different multinational companies to 455 million users located in 73 countries. The analysis provides information about the relative importance of the variables that influence individuals’ decisions to open a promotional e-mail and shows that the frequency of mailing and the use of segmentation techniques significantly affect the individual’s attention to e-mail marketing communications. The results also show a non-transparent opportunity cost associated with every e-mail sent and give advice on how to control that virtual cost. The research provides further recommendations to marketing professionals to improve the effectiveness of e-mail marketing campaigns.

## Introduction

By 2021, around 52 percent of the world population was expected to have access to e-mail services (Radicati 2019). In developed countries, this number is much higher; for instance, the number of e-mail users was expected to reach 80 percent of the US population by 2021 (eMarketer 2020). Companies are aware that the best way to optimize both offline and online marketing channels is to create an ecosystem in which both work in tandem (Ballestar et al. 2019). Consequently, e-mail marketing has been widely adopted among companies, and more than 90 percent of the top 500 online retailers in the United States have an e-mail marketing program (Heiens and Narayanaswamy 2016). E-mail marketing, or advertising through promotional e-mails, has become a key activity for digital business, including start-ups (Schmengler and Kraus 2010), driving US and UK companies to invest 8.2 percent of their digital marketing budget in e-mail marketing in 2019 (Gartner 2019). Despite the emergence of other digital media with promotional purposes, such as social media, e-mail marketing is still one of the most profitable marketing techniques (Bawm and Nath 2014) in terms of return on investment (ROI), with reported median ROIs of 122 percent (eMarketer 2016) and estimated average revenue of £42 for each pound spent (DMA 2019). To provide an idea of the prevalence of e-mail marketing, in the summer of 2020, a majority of the marketers at major UK and US brands increased their spending on e-mail marketing, even with lockdowns motivated by the COVID 19 pandemic heavily impacting marketing budgets (eMarketer 2020). All these data points highlight the importance of e-mail marketing as a fundamental communication channel for businesses, due to its widespread adoption and its above-par revenue-generating capabilities.

Due to the relevance of e-mail marketing, there is increasing interest from the corporate world in researching its effectiveness (Gartner 2020), especially given its ability to transform users’ value perceptions into loyalty (Hänninen and Karjaluoto 2017). When advertisers send a promotional e-mail, they aim to increase brand awareness, ensuring that consumers consider a given product among their different options to fulfill a specific need, stimulating the completion of a transaction and improving loyalty (Mullen and Daniels 2009). Specialized consultants develop global studies to understand how to better achieve those objectives (Gartner 2020), and even ‘small’ advertisers have been conducting A/B/n tests for years to optimize campaign results (Bonfrer and Drèze 2009). However, academia has devoted little attention to e-mail marketing and its effectiveness (Biloš et al. 2016) and, in general, research on e-mail marketing lacks a solid theoretical basis (Sigurdsson et al. 2013) and is focused on empirical studies (Hartemo 2016). In addition, existing research on e-mail marketing effectiveness is highly heterogeneous (Biloš et al. 2016) and has focused on reduced sets of variables or used limited and homogeneous samples, which hinders the generalizability of results and provides limited insight. Hence, there is relative consensus that more research is needed on this topic (Waheed and Jianhua 2018).

This lack of literature is particularly striking when we consider the challenges that companies face in the current global, hyper-competitive, and digital context. In a world saturated by promotional messages, it is becoming increasingly difficult to capture the attention of users. Further, in contrast to other media that show the entire message to the consumer directly, e-mail marketing requires that users perform a certain behavior—opening the mail—in order to have access to its content, which hampers the effectiveness of e-mail marketing campaigns. As an example, in the first half of 2019 recipients only opened 22 percent of the promotional newsletters they received (GetResponse 2019).

Finding the best way to attract consumers’ attention would dramatically improve the effectiveness of e-mail marketing communications. Therefore, the main goal of this exploratory study is to identify the variables that may influence a recipient’s decision to pay attention to promotional e-mails and to assess their relative importance. This research aims to address key methodological gaps—existing studies have focused solely on a reduced number of e-mail variables—and sample limitations—the vast majority of studies on e-mail marketing effectiveness have only focused on one country (Lorente-Páramo et al. 2020b)—by performing an extensive literature review to incorporate all variables with potential influence on user’s attention and by determining their relevance using a sample of real campaigns sent by tourism and hospitality advertisers in 73 countries.

## Background

### Attention and opening effectiveness in e-mail marketing

Attention is “the act or state of applying the mind to something” (Merriam-Webster 2021) and it plays a key role in marketing effectiveness (Moriarty 1983). Because attention is a limited cognitive resource, consumers consciously select the stimuli that merit their attention based on available perceptual information (Kahneman 1973). Advertisers that are aware of this limitation devote significant resources to capturing the attention of consumers through careful selection of sticky tunes, disruptive images and memorable messages (Solomon et al. 2013) with the objective of “catching their eye” so that they consume the advertising content (Gazizova et al. 2020). This makes attention a “physiological measure of consumer engagement” (Yang et al. 2020).

There is an ever-increasing mismatch between attention supply and attention demand caused by multiple factors such as multitasking, multiscreening, or increasing numbers of brands and products to advertise (Santoso et al. 2020). Paying attention is not an dichotomous activity, as it can be provided in different degrees (Maclnnis and Jaworski 1989). However, fortunately for advertisers, initial evidence suggests that digital advertising is effective despite low attention (Santoso et al. 2020).

Given that consumers require an initial stimulus to determine whether a particular piece of advertising merits attention, in the context of e-mail marketing we can expect this process to occur when users scan their inbox, since this is the first moment at which subscribers are exposed to a promotional e-mail (we consider notifications of new e-mails on mobile phones as an extension of the inbox, as they present the same information to consumers). While empirical determination of attention is a challenging task on most advertising channels, the particularities of e-mail marketing makes this job simpler: because of clutter, less relevant e-mails tend to be deprioritized and remain unopened (Magee 2013). Therefore, attention can be confirmed if the recipients open an e-mail because the action of opening the e-mail demonstrates willingness to “apply their mind” to that particular communication. This is consistent with previous research on digital media that considers that attention is elicited in the very first interaction between consumer and advertising (Goodrich 2011).

Considering that effectiveness refers to the “degree to which something is successful in producing a decided, decisive or desired effect” (Merriam-Webster 2021), the “opening effectiveness” of a promotional communication may be defined as “the degree to which advertisers are successful in having subscribers open promotional e-mails”. This metric assesses the ability of a promotional e-mail to trigger an initial but highly important behavior on the recipient, because opening the e-mail represents a first interaction between the advertiser and the consumer and it means that the communication has most likely captured the user’s attention (Arnold 2008).

Opening effectiveness can be measured through open rates (OR), a ratio that determines the percentage of sent e-mails that have been opened (Bonfrer and Drèze 2009; San-José-Cabezudo and Camarero-Izquierdo 2012; Andersson et al. 2014; Balakrishnan and Parekh 2015; Lorente-Páramo et al. 2020b, 2021). E-mail service providers may deliver information about open-rates to advertisers; for example, by counting the number of requests to the server that stores some visual or other, sometimes hidden, elements of the e-mail (Lim et al. 2016). This allows evaluation of the overall performance of a certain e-mail sent through the analysis of the e-mail features (length, topic, etc.). However, an analysis that incorporates the recipient’s sociodemographic features (gender, age, job, etc.) requires advanced tracking and analytics capabilities that make it possible to identify the specific behavior of each recipient (that is, who opened the email and who did not) and detailed sociodemographic information from each recipient that is difficult to collect without impacting the overall performance of the email marketing program (Groves 2009). Given that the present research focuses on reaching the broader possible sample from email programs of different size and complexity, independent variables will not include sociodemographic data from recipients, to avoid limiting the sample to companies with advanced analytics capabilities and rich subscriber databases.

The investigation of opening effectiveness involves the evaluation of the moment consumers access their inbox, as well as the multiple circumstances that may influence their decision to open a new e-mail. Given the great diversity and number of elements that can influence the decision to open an e-mail, it would be beneficial to use a comprehensive framework to structure the analysis. Based on previous literature on attention and e-mail effectiveness, this research differentiates between visible (both formal and content-related), temporal and contextual variables, an approach similar to that followed by previous studies of e-mail marketing performance (Lorente-Páramo et al. 2020a).

The relevance of visible elements of the e-mail—that is, sender and subject line—is straightforward because they contain all the information available to the user at that stage (Micheaux 2011). Similarly, because the recipient’s mood and amount of time available might differ greatly depending on when the user receives the e-mail (for example, before, during or after work, a workday or the weekend), the date and time the e-mail was sent might affect the recipient’s assessment of the relevance of the message and may therefore also play a role in opening effectiveness (Ellis-Chadwick and Doherty 2012). The number and frequency of e-mails previously delivered by the same sender is also important, as users are more prone to ignore the communications when they perceive that the company is too persistent (Haq 2009). Finally, contextual considerations such as tailoring the promotional messages to particular characteristics of each subscriber tend to increase the perceived relevance of the content and, consequently, the likelihood of consumers opening e-mails (Bawm and Nath 2014)*.*

### Visible elements of e-mail marketing

Visible elements are the parts of the message that are apparent in the inbox before users take any further action. Therefore, they are the only data available for the recipients to evaluate the relevance of an e-mail when they receive it. Although the number and richness of these elements may depend on the capabilities of the recipient’s e-mail client, the two main visible elements that are always present are the subject line and the sender’s name or e-mail address; both influence a user’s decision to open a promotional e-mail (Balakrishnan and Parekh 2015).

The subject line summarizes the objective of the e-mail and anticipates its content, allowing users to make a first assessment of their interest (Baggott 2011). If the subject line is unclear or appears to be irrelevant, the communication with users will stop at that point. However, if the subject line fosters curiosity or interest, there will be a chance that the recipient engages with the message (Arnold 2008). This binary operation highlights the importance of the subject line as a key determinant of e-mail marketing effectiveness (Chittenden and Rettie 2003). Despite some contrarian evidence against the relationship between subject line and opening effectiveness (San-José-Cabezudo and Camarero-Izquierdo 2012), more recent research confirms the effect of the subject line in the recipient’s decision to open an e-mail (Theerthaana and Sharad 2014; Mogos and Acatrinei 2015), with the subject line being 3.8 times more effective than the sender when it comes to influencing the decision to open an e-mail (Micheaux 2011), which is why the present study focuses on the former. Furthermore, there are two additional considerations when observing the subject line. From a purely formal perspective, it is possible to analyze its length and the presence—or absence—of special characters. From a semantic perspective, it is important to analyze the content and the meaning of the message that the advertiser tries to convey.

The relevance of formal aspects lies in the fact that the inclusion of numbers or uncommon special characters highlights the promotional nature of an e-mail. This inclusion increases the chances of capturing the recipients’ attention if and when they are considering the purchase of a certain product (Solomon et al. 2013), even though an excess of special characters can lead to automatic inbox filters categorizing the e-mail as spam (Arnold 2008). Regarding message length, long texts require a larger attention span and are therefore less effective for advertising purposes because consumers are more likely to ignore them (Solomon et al. 2013). This effect can be heightened by the recent increase in media multi-tasking, which has resulted in more diffuse attention and deterioration of ad-processing (Duff and Segijn 2019). In e-mail marketing research, the length of an e-mail has an effect on click-through rates (Lorente-Páramo et al. 2020a) and some attribution-scoring-based models are able to better predict opening effectiveness by incorporating additional elements to the model (such as the length of the subject line or the presence of special characters or numbers) due to their effect on the perceptual selection process of users (Balakrishnan and Parekh 2015). Therefore, we propose the following hypotheses:

#### H1:

The increase in the number of characters in the subject line has a negative relationship with the users’ attention to promotional e-mails.

#### H2:

The presence of numeric characters in the subject line has a positive relationship with the users’ attention to promotional e-mails.

#### H3:

The presence of special characters in the subject line has a positive relationship with the users’ attention to promotional e-mails.

From a semantic view, the goal of a well-crafted subject line is to convey a message that captures consumers’ attention and entices them to open the e-mail. Therefore, messages should not only be relevant, but also interesting enough to capture the recipient’s attention (Kumar and Salo 2018). This is why the subject line usually summarizes the content of the e-mail or highlights the main promotional ideas. Additionally, certain advertisers use disruptive approaches to stand out and differentiate from other e-mails by creating suspense, using catchphrases or other techniques to increase the relevance of the message, such as identifying a communication as promotional (Micheaux 2011), informative, or entertaining (Lu et al. 2007). Consumers may also subscribe to commercial newsletters to keep abreast of events, contests, new products, and loyalty programs, but they may show different levels of interest in each of these topics (Carmen and Nicolae 2010). Hence, it seems logical that users also present different levels of attention depending on the content of the communication. However, there is no evidence from prior research about the performance of different types of content—only Biloš et al. (2016) tested the differences between what they termed “generic” and “newsletter-specific” e-mails, so it is difficult to advance a causal hypothesis. Therefore, the following correlational hypothesis can be posited:

#### H4:

There is a relationship between the content of the subject line and users’ attention to promotional e-mails.

### Temporal elements in e-mail marketing

Besides the content of the commercial or promotional communication, determining the right moment at which the message reaches the recipient’s inbox is critical with regard to its effectiveness (Barnes 2002). For example, the relevance of a nighttime entertainment offer will likely be higher if the message is received on a Friday than on a Monday; likewise, individuals’ willingness to interact with advertising content may be higher when they are idle or using some free time (for example, while travelling on public transport) than on busier occasions.

More specifically, the day of the week and time (hour and minute) at which the message is delivered are traditionally considered important elements to optimize advertising campaigns, given their influence on the responsiveness of consumers to advertisements (Barnes 2002). There is a shared certainty among e-mail marketers that these two variables have an effect on opening effectiveness (Ellis-Chadwick and Doherty 2012; Paralič et al. 2020) and that ignoring them may lead to worse results (Groves 2009; Baggott 2011). Further, prior research confirms the effect of both variables in the effectiveness of television commercials (Tellis et al. 2000) and paid search advertising (Rutz and Bucklin 2011).

However, there is an important difference between e-mail marketing and television commercials or paid search advertising: the asynchronous nature of e-mail marketing. The fact that e-mail marketing is an asynchronous means of communication means that the moment the newsletter is sent is not necessarily the moment at which subscribers consume the content. While e-mail marketers have tools to determine the precise moment a subscriber opens an e-mail, they cannot establish the moment a user checks the inbox and decides whether to open an e-mail. Thus, the only data available is the time the message was sent. Fortunately, checking one’s inbox is often the first activity that most users perform when they connect to the Internet, and most of them do it several times a day (Faught et al. 2004). Together with the pervasiveness of always-on smartphones, it is safe to assume that there are only a few hours, if not minutes, of difference between the time an e-mail is sent and the moment at which users evaluate whether the message deserves their attention. Therefore, the day of the week and time of sending is a reasonable approximation to the moment consumers check their inbox. As in the case of content type, it is difficult to advance causal hypotheses in this regard, so we propose the following correlational hypotheses:

#### H5:

There is a relationship between the day of the week a promotional e-mail is sent and the users’ attention to that e-mail.

#### H6:

There is a relationship between the time at which a promotional e-mail is sent and the users’ attention to that e-mail.

Finally, sending frequency—the number of e-mails sent to a consumer in a given period of time (Micheaux 2011)—is another temporal element that might affect the effectiveness of promotional communications. Sending frequency is considered of paramount importance in e-mail marketing (Baggott 2011) for two reasons. On the positive side, a higher number of sent newsletters increases the chances of getting one of them opened by users. On the negative side, shorter sending periods reduce the value of the communication (Haq 2009), to the point that users might decide to cancel their subscription to the database (Groves 2009) or move all future e-mails to the “spam” folder (Kimixay et al. 2019) if they consider that a company is flooding their inbox with irrelevant e-mails.

While an increase in frequency does not worsen subscribers’ attitudes towards e-mail marketing (Haq 2009), e-mail marketers are concerned about the possibility that it may cause irritation in the recipients (Ellis-Chadwick and Doherty 2012). Sending frequency plays a role in the consumer’s perception process, and more frequent e-mails may trigger tedium (Tellis et al. 2005) or adaptation to the perceptual stimulus (in other words, the recipients may determine that the most recent stimuli do not contain any new information, and therefore do not merit their interest), which in turn reduces the effectiveness of the communication (Solomon et al. 2013). Thus, the following hypothesis is posited:

*H7:* An increase in sending frequency of promotional e-mails has a negative relationship with users’ attention to promotional e-mails.

### Contextual elements in e-mail marketing: segmentation

Before the emergence of digital marketing, advertisers struggled to find the right balance between reach and audience segmentation. Traditional communication media facilitate higher reach of commercial communications, but have evident limitations when it comes to tailoring the content of the message to different audiences (Barnes 2002). For example, an advertisement aired during the break of a TV show reaches the viewers directly and in a simple way, but the content of the promotional communication is the same for all of the viewers. Aware of this limitation, major US television networks have started working in personalized advertising sales platforms (Ng 2015), but these are currently exceptions to the norm, and the results of these efforts are yet to be seen.

Despite some exceptions that facilitate certain geographical flexibility, such as outdoor advertising on bus stops, the majority of traditional channels used for advertising generally lack the means to adapt and customize the message to the recipient based on sociodemographic data. Consequently, advertisers plan media campaigns by matching their product’s target consumers with advertising spaces whose audiences are closer to that group of target consumers. Examples include using the profile of the average viewer of a particular TV show, or analyzing the characteristics of residents of different neighborhoods to determine where to launch an outdoor advertising campaign (Yancey et al. 2009). While these techniques increase the affinity of messages and audiences, a high number of advertising impacts will surely remain irrelevant.

The possibility of tailoring advertisements to certain groups of consumers (segments) based on a wide range of variables is one of main advantages of the use of digital media for commercial communications (Baggott 2011). In this regard, e-mail marketing provides higher flexibility because it is based on personal data by design: when a user subscribes to a newsletter, the company usually requests some socio-demographic information. Additionally, the possibility of recording users’ interactions with previously sent e-mails facilitates the collection of implicit information about their interests, which can be extremely valuable for improving the results of future campaigns (Jackson and DeCormier 1999; Bawm and Nath 2014). Thus, the following hypothesis is posited:

#### H8:

Sending promotional e-mails that are tailored to the characteristics of specific user segments has a positive relationship with users’ attention to those e-mails.

**Fig. 1:**
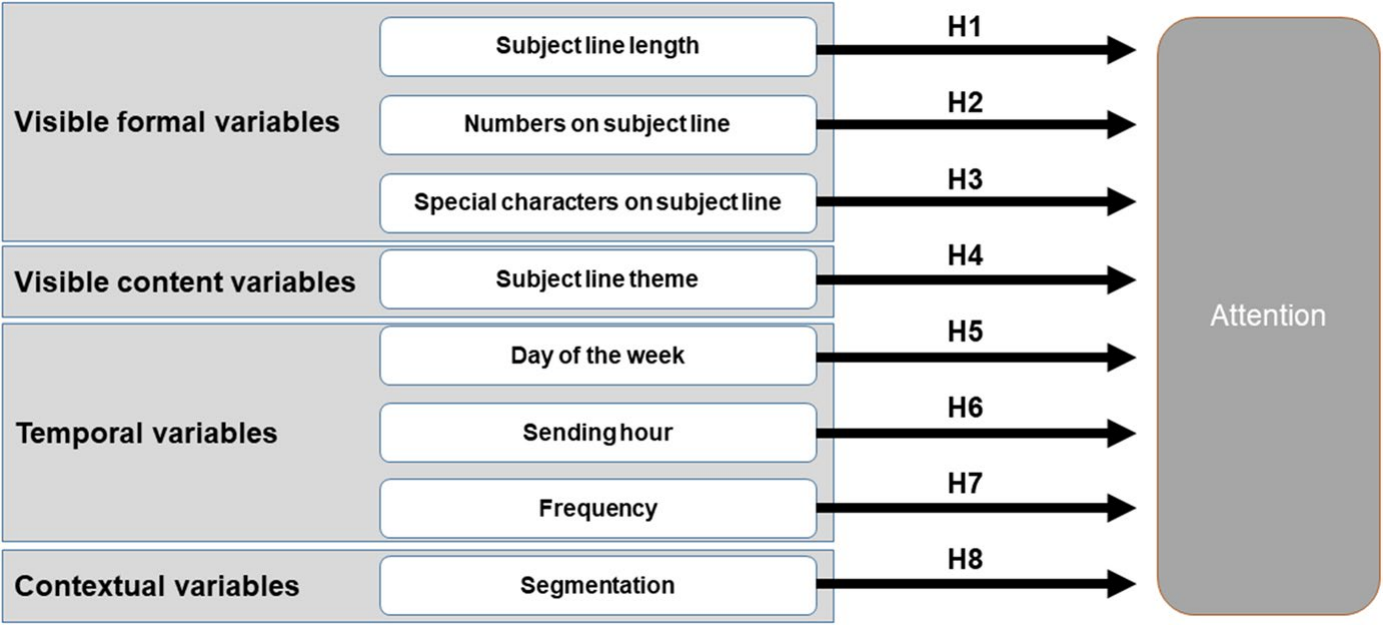
Summary of research hypotheses

Figure 1 summarizes the theoretical model and research hypotheses proposed to evaluate the variables of influence in the attention paid to promotional e-mails.

## Methodology

### Sample of the study

In order to determine the right sample for this research, it is worth considering that the product category may influence the effectiveness of online advertising (Shamdasani et al. 2001), which involves that different industries may have different average open rates (Biloš et al. 2016; GetResponse 2019; eMarketer 2020). Hence, the empirical data gathered focuses on a single product category (tourism and hospitality), and includes companies such as airlines, hotels, and airports. This industry is especially suitable for this study because its international nature means that most companies in the industry have customers from multiple countries, which makes it relatively easy to obtain geographically diverse samples from e-mail marketing programs of a small number of comparable players. The basic data unit used in the analysis is the marketing campaign, each of which comprises all the promotional messages sent to the subscriber database. The dataset, obtained from several sources including GetResponse (an e-mail service provider) comprises 5765 different promotional e-mails sent between 2013 and 2018 by several multinational companies to 455 million users (who provided consent to receive the communications) across 73 countries. The global nature of the sample is one of the key differentiating factors of this study, but it comes at the cost of generating potential issues regarding cross-country equivalence. While we have addressed some of the potential issues by smart operationalization of variables (for example, by adjusting the day of the week to account for the different beginning of weekends by country), we have not looked at more subtle differences among countries, such as the frequency threshold beyond which an advertiser is considered too persistent.

### Variable operationalization

The operationalization of formal visible variables addresses the most relevant aspects of the subject line: length (total number of characters, including spaces and punctuation marks (Balakrishnan and Parekh 2015)) and inclusion of numbers and special characters (both defined as dichotomous variables with a value of 1 if numbers or special characters are present, and 0 if not). Special characters do not include the most common punctuation marks (in particular:,.;:’) because the main objective is to capture and analyze distinctive elements and punctuation marks are present in most sentences in some languages. After observing and analyzing the collected data, we found the following special characters and included them in the study: ! ‘$€%&/()?¡^` + *¨'-_ <  > …-•

The content of the subject line was determined using a semantic analysis to classify and assign each e-mail to one of the categories defined by Ellis-Chadwick and Doherty (2012) (Table 1): discount/saving, product detail, newsletter, seasonal promotion, teaser, action prompt, sale, contest, in-store event, free gift, bonus offer, and others. Having observed cases that could belong to more than one category, we added two additional categories to the original proposal: seasonal discounts and sale with contest. All other cases that could belong to more than one category were not numerous enough to merit a specific category, so they were classified as “other”.

**Table 1:** Classification of subject lines by content type

Type	Criteria
Discount/saving	Promotion explicitly mentioning a reduction in the regular price (e.g., “20% discount” or “10€ discount”)
Seasonal promotion	Promotion that does not belong to the previous category and has a theme related to a particular season (e.g., Summer, Fall), special days (e.g., Valentine’s Day, Black Friday), religious festivities (e.g., Christmas, Hannukah) or National Holidays (e.g., Independence Day)
Seasonal discount	Any promotion belonging to both previous categories
Free gift	Promotion explicitly mentioning a free gift
Bonus offer	Promotion offering additional points on loyalty cards or offering virtual currency for loyalty programs
Sale	Any promotion not included in any of the previous categories
Teaser	E-mail announcing a future campaign
Action prompt	E-mails prompting an action from the user that is different from a purchase or participation in a contest
Product details	Informative e-mail related to a particular product
Newsletter	Informative e-mail related to any other topic (i.e., unrelated to a product)
Contest	Contest-related e-mail (e.g., invitation to participate, T&Cs, winners)
Sale with contest	Same as above, but including additional information about a sale
In-store event	E-mail enticing users to visit a physical store
Other	E-mails not belonging to any of the previous categories

Regarding frequency, previous research on the effectiveness of banners or TV ads has defined this variable as the number of times a specific ad has been shown to the user (Broussard 2000; Tellis et al. 2000). This definition cannot be easily translated to e-mail marketing because users expect to receive different e-mails every time, as opposed to watching the same advertisement on TV multiple times. To address this problem, we propose a similar approach to the one used in RFM (recency, frequency, monetary) pattern models (Miglautsch 2000; Chen et al. 2009) and define the frequency of a particular e-mail as the number of e-mails sent by the company to the same user in the previous 30 days. Other temporal variables, such as day of the week and sending time, were determined based on the local time of the country in which the majority of subscribers are based (mailermailer 2016).

Additionally, we consider that an e-mail has been tailored or segmented when it has been sent to a limited portion of the database that has been selected according to criteria based on personal preferences—such as a particular language in multilingual countries (O’Guinn et al. 1985)—or consumer habits—such as interest in a given product (Sigurdsson et al. 2016). This information has been coded as a dichotomous variable named ‘segmentation’. After reviewing the whole sample, all records have valid values for all the variables. Table 2 summarizes the sample details.

**Table 2:** Descriptive statistics of the sample

Variable type		Variable	N	Var = 1	%	Var = 0	%
Visible	Formal	Length	5765				
		Numbers	5765	3888	67	1877	33
		Special characters	5765	4081	71	1684	29
	Content type	Discount/saving	5765	1984	34	3781	66
		Seasonal promotion	5765	323	6	5442	94
		Seasonal discount	5765	61	1	5704	99
		Free gift	5765	10	0	5755	100
		Bonus offer	5765	38	1	5727	99
		Sale	5765	2474	43	3291	57
		Teaser	5765	0	0	5765	100
		Action prompt	5765	0	0	5765	100
		Product details	5765	294	5	5471	95
		Newsletter	5765	94	2	5671	98
		Contest	5765	135	2	5630	98
		Sale with contest	5765	29	1	5736	99
		In-store event	5765	0	0	5765	100
		Other	5765	323	6	5442	94
Time-related		Frequency	5765				
		Day of the week	5765				
		Time sent (hour of the day)	5765				
Contextual		Segmentation	5765	172	3	5593	97

Finally, the dependent variable (attention to promotional e-mails) is measured by open rates.

## Results

As Table 2 shows, there were no records classified as “teaser”, “action prompt,” or “in-store event”, so the final number of content categories (types of promotional messages) is 11. This nominal variable has been operationalized through 10 dummy variables that reflect the difference of the linear model versus “sale”, the most frequent category. Analogously, the variables “day of the week” and “time sent (hour of the day)” have been entered in the model using as reference values “Tuesday” and “9:00 a.m.”, the most frequent categories.

The data analysis involves ordinary least squares linear regression, a method commonly used in the analysis of the effectiveness of digital media (Baltas 2003; Robinson et al. 2007). We used the forced entry method to avoid any sort of researcher bias (Pedhazur 1997). The initial analysis detected multicollinearity issues with the variable “time sent”, which was subsequently excluded from the analysis, and therefore H6 could not be tested. To avoid lack of normality of the dependent variable, which showed kurtosis and skewness issues, the analysis applied a logarithmic transformation (Robinson et al. 2007). After the transformation, the existence of extreme values and influential cases was ruled out upon inspection of Cook’s distance (Cook and Weisberg 1982). Having removed the “time sent” variable, we discarded multicollinearity issues after observing the VIF values; normality of the residuals was verified after observation of the P-P plot; and homoscedasticity and linearity were confirmed after the analysis of the scatterplot of standardized residuals-standardized predicted values (Field 2013).

The final model is significant (F = 48.104, sig. = 0.000) and explains 15 percent of the variance (R^2^ = 0.150) in open rates. The intercept is significant at 3.002, so the open rate of a typical e-mail is 20.13 percent, slightly under the average figures in industry reports (GetResponse 2019) and previous experiments (Biloš et al. 2016). Table 3 summarizes the results of the analysis.

**Table 3:** Results of the data analysis

Variable type		Variable	*β*	Sig	B [conf. int.]	Std. Error
﻿Intercept		Intercept		3.002	.020	
Visible	Formal	Length	.011	.378	.000	.000
		Numbers	.017	.241	.014	.012
		Special characters	.050***	.000	.043 [.019, .067]	.012
	Content	Discount/saving	− .135***	.000	**− **.110 [**− **.133, **− **.087]	.012
		Seasonal promotion	.066***	.000	.111 [.069, .152]	.021
		Seasonal discount	**− **.018	.155	**− **.067	.047
		Free gift	.035**	.004	.329 [.104, .553]	.115
		Bonus offer	− .033**	.007	**− **.158 [**− **.273, **− **.043]	.059
		Product details	.021	.108	.036	.023
		Newsletter	.025*	.043	.078 [.002, .153]	.038
		Contest	**− **.014	.265	**− **.035	.032
		Sale with contest	-.006	.617	**− **.034	.067
		Other	.080***	.000	.134 [.091, .177]	.022
Time-related	Frequency	Frequency	− .252***	.000	**− **.065 [**− **.071, **− **.058]	.003
	Day of the week	Friday	− .056***	.000	-.058 [**− **.087, **− **.029]	.015
		Monday	.044**	.001	.063 [.026, .100]	.019
		Saturday	− .052***	.000	-.064 [-.098, -.031]	.017
		Sunday	**− **.013	.293	**− **.069	.065
		Thursday	.017	.231	.018	.015
		Wednesday	− .540***	.000	**− **.054 [**− **.082, **− **.025]	.014
	Time sent	Hour of the day	Excluded			
Contextual		Segmentation	.222***	.000	.506 [.450, .562]	.029

The significant bold value indicates *p* < 0.05

Confidence interval estimated as 95 percent confidence interval for B (only shown for significant relations)

^*^p < 0.05; **p < 0.01; ***p < 0.001

Effect sizes were assessed by removing significant predictors and observing the reduction of R^2^ (Trusty et al. 2004). Those results are presented in Table 4.

**Table 4:** Effect sizes of significant predictors

Variable	R^2^ reduction after variable removal (Original R^2^ = 0.15)
Frequency	0.061
Segmentation	0.047
Content (semantic)	0.030
Day of week	0.011
Special characters	0.005

The analysis also includes the study of interaction effects among variables to rule out the effect of specific combinations. More precisely, the analysis tested the following interaction effects:

- Whether formal variables may have interaction effects between them. The analyzed interactions include the presence of numbers and presence of special characters (Nums*SpecChars), the presence of numbers and subject length (Nums*SubjLength), and the presence of special characters and subject length (SpecChars*SubjLength).
- Whether the presence of numbers on the subject line of quantitative-themed promotions has an effect on performance. The potential interactions consider the two predominant types of e-mail and therefore analyze the interactions between numbers and discount/savings e-mails (Nums*DiscSav) and numbers and seasonal promotions (Nums*SeasProm).
- Whether the length of e-mails may have a negative influence on the effect of high sending frequency; that is, the interaction effect between frequency and subject length (Freq*SubjLength).
- Whether higher frequency affects performance of the two most frequent content types: interaction between frequency and discount/savings e-mails (Freq*DiscSav), and between frequency and seasonal promotions (Freq*SeasProm).
- Whether the negative effect of frequency of sending is reduced when e-mails are segmented because they are better tailored to the recipient’s needs and interests —interaction between segmentation and frequency of sending (Seg*Freq).
- Whether discount promotions are more effective on segmented than on non-segmented e-mails; that is, interaction between segmentation and discount/savings e-mails (Seg*DiscSav).

Out of the 10 interactions tested, only the pairs Nums*SubjLength and Freq*DiscSav were significative, albeit only contributing to a marginal improvement on the variance explained (F = 45.359, sig. = 0.000, R^2^ = 0.154). Table 5 shows the results of the simple slopes analysis (Aiken et al. 1991) conducted to determine the direction of the interaction.

**Table 5:** Analysis of (significant) interaction effects

Subject length * Presence of numbers in subject (Nums*SubjLength)	Presence of numbers	b (effect of subject length on open rate)	t-value	p-value
	No	− 0.0014	− 3.0241	0.0025
	Yes	0.0009	3.0472	0.0023

Frequency of sending * Discount/savings (content type) (Freq*DiscSav)	Discount/savings	b (effect of frequency on open rate)	t-value	p-value
	No	− 0.0566	− 14.7086	0.0000
	Yes	− 0.0795	− 14.1958	0.0000

**Fig. 2:**
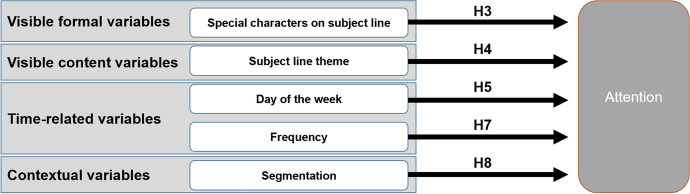
Summary of supported hypotheses

Figure 2 summarizes the hypotheses supported by the empirical analysis of attention paid to promotional e-mails.

## Discussion

This research makes a relevant contribution to the study of effectiveness in e-mail marketing along the visible, temporal, and contextual dimensions of the phenomenon. As this section shows, the results of the study provide cues to digital marketers to improve the performance and relevance of their campaigns, and open interesting avenues of research for academics. From the perspective of this journal, the study also enriches current research by exploring themes beyond core management topics, such as corporate social responsibility (Mas-Tur et al. 2020)﻿.

The results suggest that there is no direct relation between the length of the subject line and the attention paid to promotional e-mails, rejecting H1; this finding contrasts with the significant relationship found in the effectiveness of web banners (Baltas 2003; Robinson et al. 2007) and with the idea that individuals require greater effort to process long texts (Solomon et al. 2013), but it is in line with previous industry reports (Stallings 2009) suggesting that additional information in the subject line may be appreciated by some audiences, especially highly targeted ones. Nonetheless, we cannot discard the potential influence of message truncation: some e-mail service providers truncate the subject line based on screen width; for example, the number of characters shown varies between 27 and 64 characters across different mobile operating systems and screen resolutions (Stiglitz 2015). Because more than half of promotional e-mails are opened in mobile phones (Mailermailer 2016), a significant number of subscribers may be using only the first few characters of the subject line to evaluate their interest on an e-mail.

The presence of numbers in the subject line does not seem to be related to open rate either, rejecting H2, but the inclusion of special characters seems to have a positive and significant relationship with attention (that is, open rate), supporting H3. Interestingly, a closer look to the data reveals that 67 percent of the promotional e-mails include numbers, nearly the same percentage of e-mails that include special characters. This finding, which merits further investigation, might suggest that numbers might no longer be considered a distinctive element, but special characters might still have a disruptive component that facilitates capturing the recipient’s attention.

The interaction analysis (Table 5) suggests a marginal but significant interaction between the presence of numbers and subject length, suggesting that subject length might slightly improve open rates with the presence of numbers in the subject line, again supporting the idea that the inclusion of numbers may provide additional information that users consider valuable. However, the marginal improvement of the variance explained suggests the need for further investigation of this interaction effect.

Regarding the type of content of the subject line, the results support H4. It is particularly surprising that the addition of a discount to a generic promotional communication seems to reduce its effectiveness. The inclusion of economic incentives is a recurrent advertising technique (Kotler and Keller 2012) with proven effects on other online advertising channels (Ballestar et al. 2019). Economic incentives aim to leverage the cognitive components of attitudes’ vulnerability to persuasive arguments (Solomon et al. 2013), an approach that usually increases advertising effectiveness when consumers are involved with the product category (Petty et al. 1983). However, the results suggest that consumers are less receptive to discounts and bonus offers than to generic sales and that they prefer seasonal offers or free gifts to generic sales. In addition, recipients also value other types of e-mail promotional communications from the company. Regarding non-promotional communications, only newsletters receive more attention from users than generic sales. The lack of effectiveness of economic incentives contests both empirical (Biloš et al. 2016) and non-empirical (Chang et al. 2013) studies that propose this technique as a way to improve performance of e-mail marketing campaigns, and is a key contribution of this research, given the consensus on its effectiveness in other e-mail marketing metrics, such as click-through rate (Rettie and Chittenden 2003; Sigurdsson et al. 2016) and conversion rate (Sigurdsson et al. 2013; Theerthaana and Sharad 2014), as well as its widespread use among consumer goods companies (Utkarsh and Gupta 2019). It is important to note that the result found in this study is consistent with field research on consumer goods, which shows that store promotions that do not explicitly mention discounts (such as bundling offers or free samples) are more effective than discounts (Tello and Zamora 2014), and suggests that this offline trend may also applicable to the study of attention on e-mail marketing.

The results also confirm H5. The day of the week the message is sent seems to be significantly related to effectiveness, with Monday apparently being the day with the highest effectiveness and Wednesday being the least effective. The confirmation of the relevance of the day of the week the communication is sent is another contribution of this investigation, which includes e-mail marketing in the list of communication media that are influenced by weekday, same as paid search or TV.

As expected, the increase of sending frequency has a negative influence on open rate, supporting H7. From the analysis of interaction effects, the negative influence is augmented in the case of e-mails dealing with discount or savings. Along with segmentation, sending frequency exerts the highest influence on attention to promotional e-mails. Therefore, both variables should be the top priority for practitioners aiming to improve the performance of their e-mail marketing campaigns. This finding contrasts with the minimal attention that sending frequency has received so far in academic research on e-mail marketing. While there is no general rule on what might be the right frequency, as the preferred levels vary by segment (Baggott 2011), this finding helps support the recommendation of carefully considering the potential benefit of each new e-mail against the inconvenience that it might generate (Dufrene et al. 2005).

Regarding segmentation, the results suggest a positive and significant relationship between this variable and the recipient’s attention to promotional e-mails, secondary only to sending frequency, and supporting H8. This finding is in line with the positive perceptions of practitioners about personalization and current digital marketing trends that emphasize the importance of delivering personalized content to enhance customer experience (AMA et al. 2017). For example, something as simple as addressing the recipient by his or her name may significantly improve open rates (Sahni et al. 2018). As a note of caution, the use of relevant criteria for the creation of segments and the design of specific content adapted to each of them are necessary foundations to ensure the success of this technique. Some examples of adequate variables to build segments are those that help identify target consumers –sociodemographic, geographic, values, etc. (Baggott 2011; Madi 2016)—those recorded from interactions with previous e-mails (Bawm and Nath 2014; Key 2017) and those related to other touchpoints with consumers, such as loyalty programs or data from CRM systems (Belch and Belch 2003). Beyond simple segmentation approaches, more complex techniques such as RFM scoring of subscribers are now relatively widespread among organizations that want to improve their performance (Buruncuk and Badur 2010), and new techniques based on the application of artificial intelligence and machine learning are preparing the new wave of personalized e-mail content delivery. This requires synchronization among multiple areas of the company (IT, sales, marketing, e-commerce, etc.), an objective that might be more difficult to attain for traditional companies than for pure online players (de Groote et al. 2020).

### Managerial implications

The results of the empirical study provide marketers with valuable insights into the main variables of interest and their influence when the goal is to increase the attention that users pay to their promotional e-mails. The analysis of effect sizes (Table 4) suggests that marketers’ efforts should be directed towards reducing sending frequency and using segmentation techniques, the actions that can have a greater impact on open rates. Effect sizes range between 0.01 and 0.06; even though they may be considered ‘small’, Sahni et al. (2018) noted that, in general, ad impressions have small effect sizes.

The negative relationship of sending frequency and open rate (H7) suggests the existence of an opportunity cost for each e-mail sent; this cost may not be cancelled out by subsequent e-mails, which are likely to have lower effectiveness. Contrary to other digital media based on pay-per-click or impression models, e-mail marketing campaigns have had a negligible cost so far; however, and considering this opportunity cost, advertisers must be selective when implementing their e-mail marketing campaigns, applying the same scarcity mindset used in other channels where budget limitations act as catalyst for a careful selection of messages.

The creation of a virtual cost mechanism may be helpful for measuring and controlling the opportunity cost of each promotional e-mail sent. For example, the team responsible for e-mail marketing at a company could assign a limited number of credits per year to the different communication teams that regularly send promotional e-mails, so that every time a promotional team sends an e-mail, a credit would be deducted from their account. Additional ways to reduce this opportunity cost include the accommodation of multiple messages in a single newsletter, such as templates that contain areas for multiple stories.

Database segmentation, the other variable with key influence on performance (H8), not only allows sending personalized communications to a reduced subset of the database, helping reduce sending frequency, but also has the added benefit of increasing open rates. The double effect on performance, both direct and indirect through frequency reduction, is likely to trigger a relevant improvement of open rates. Therefore, using segmentation techniques on all communications may be the single most impactful action e-mail marketers can take to improve attention to their e-mails. Voice-of-the-customer programs that facilitate the identification of consumer archetypes are a good starting point for segmenting database users, in addition to the tactics already mentioned in the previous section. Firms with limited resources that cannot afford detailed voice-of-the-customer programs can use tactics rooted in entrepreneurial marketing with proven impact on performance, such as increasing risk-taking (Eggers et al. 2020). In the area of segmentation, this approach could be substantiated in the use of limited samples or the re-application of segmentation criteria already tested successfully in other businesses, and then assessing their performance. This approach should not be limited to start-ups, as entrepreneurial marketing tactics are applicable irrespective of firm size (Kraus et al. 2010).

On a second level of priority, variables such as content of the e-mail, the day of the week on which it was sent or presence of special characters in the subject line (H3–H5) might also influence open rates and should not be neglected by marketers. For example, lower sending frequency provides some flexibility in the selection of the day of the week the e-mails are sent, and marketers should take advantage of this flexibility to increase effectiveness by choosing the best moment to send the e-mails, preferably at the beginning of the week.

The study also provides insights into the effectiveness of the different topics covered by the subject line. As Table 2 shows, 43 percent of the promotional e-mails focus on sales and more than one-third focus on discounts or bonuses. However, and despite their prevalence, the results indicate that promotional e-mails of a quantitative nature, such as those related to discounts or bonuses, seem to be less effective at capturing the recipient’s attention. The decreased effectiveness of discount-related messages, combined with the lack of relationship between the inclusion of numbers in the subject line and open rates, might be an indication of the application of heuristic processing (Aigner et al. 2019) by recipients to cope with large volumes of promotional e-mails that focus on discounts; therefore, subscribers might actually be performing a semi-automatic filtering process where they rule out these messages and deem them not worthy of attention.

This finding warns about the perils of just focusing on promotional messages (sales, discounts, etc.) that entice subscribers to complete one-time purchases. The result is also a wake-up call for marketers and companies to explore innovative ways to craft and design their messages, aiming to stand out, engage at a personal level, and leave a lasting impression, or even resort to off-the-beaten-path themes, such as free gifts or seasonal promotions. Once again, entrepreneurial marketing can offer an answer to the conundrum marketers face regarding content; for example, popular techniques such as crowdsourcing (Alqahtani and Uslay 2020) can lead to outsourcing part of the content strategy to the subscribers by proactively seeking one-on-one conversations with the most engaged audience, to determine what they like/dislike, or by setting content creation contests for subscribers (for example, “send us your perfect newsletter and win a free product”).

### Limitations and future research

Due to multicollinearity issues, the study could not assess the impact of the hour of the day at which the promotional message is sent on the recipient’s attention to the e-mail. Because a correct mix of day of the week and hour of the promotional communication might lead to optimal combinations, further exploring this variable is a promising research line.

The results indicate that the length of the subject line does not have an influence on opening effectiveness. However, we did not control for the device used by recipients to check their e-mail. Considering the now prevalent use of smartphones, the results cannot discard the effect of automatic reduction of length of the subject line in these devices. Future research should further investigate this effect to confirm the results of this study.

The analysis finds that segmented e-mails perform better than non-segmented e-mails. However, further research is needed to better understand how to optimally implement segmentation to increase the effectiveness of e-mail marketing campaigns, making it necessary to determine which segmentation criteria are the most adequate.

The sample used for this research belongs to a single product category. Similar analysis performed with data from other industries could shed more light on the variations of consumer attitudes towards different product categories.

Finally, while the use of a global and multicultural sample is an important contribution of this study and supports higher generalizability of the results, it does so at the expense of omitting the potential direct or moderating influence of factors related to the recipient such as cultural and demographic factors (age, gender, etc.), opening a door to the inclusion of these variables in future studies, to both enrich the results and address the potential concerns about cross-country equivalence.

### Concluding remarks

This research provides information to e-mail marketers about how to improve the attention that subscribers pay to promotional e-mails by incorporating slight and inexpensive changes to their current practice. The results of the study highlight the importance of higher control over sending frequency and the need for application of segmentation techniques and personalization to increase recipients’ attention and improve opening effectiveness. Other priorities include the selection of non-quantitative topics, the inclusion of special characters in the subject line, and the delivery of personalized newsletters at the beginning of the week. Finally, the research makes a notable theoretical and methodological contribution to the study of e-mail marketing effectiveness by proposing a new categorization of influencing variables–visible, temporal, and contextual —that can facilitate future research. Such categorization could also be extended to research on effectiveness of any other digital marketing channel.

## Declarations

### Data availability

The data used in this research are not available to the public.
